# LAB/NTAL Facilitates Fungal/PAMP-induced IL-12 and IFN-γ Production by Repressing β-Catenin Activation in Dendritic Cells

**DOI:** 10.1371/journal.ppat.1003357

**Published:** 2013-05-09

**Authors:** Selinda J. Orr, Ashley R. Burg, Tim Chan, Laura Quigley, Gareth W. Jones, Jill W. Ford, Deborah Hodge, Catherine Razzook, Joseph Sarhan, Yava L. Jones, Gillian C. Whittaker, Kimberly C. Boelte, Lyudmila Lyakh, Marco Cardone, Geraldine M. O'Connor, Cuiyan Tan, Hongchuan Li, Stephen K. Anderson, Simon A. Jones, Weiguo Zhang, Philip R. Taylor, Giorgio Trinchieri, Daniel W. McVicar

**Affiliations:** 1 Cancer and Inflammation Program, National Cancer Institute-Frederick, Frederick, Maryland, United States of America; 2 Institute of Infection and Immunity, Cardiff University School of Medicine, Cardiff, Wales; 3 Department of Comparative Pathobiology, Purdue University School of Veterinary Medicine, West Lafayette, Indiana, United States of America; 4 Experimental Immunology Section, Laboratory of Immunology, National Eye Institute, National Institutes of Health, Bethesda, Maryland, United States of America; 5 Basic Research Program, SAIC-Frederick Inc., National Cancer Institute-Frederick, Frederick Maryland, United States of America; 6 Department of Immunology, Duke University Medical Center, Durham, North Carolina, United States of America; University of Massachusetts Medical School, United States of America

## Abstract

Fungal pathogens elicit cytokine responses downstream of immunoreceptor tyrosine-based activation motif (ITAM)-coupled or hemiITAM-containing receptors and TLRs. The Linker for Activation of B cells/Non-T cell Activating Linker (LAB/NTAL) encoded by *Lat2*, is a known regulator of ITAM-coupled receptors and TLR-associated cytokine responses. Here we demonstrate that LAB is involved in anti-fungal immunity. We show that *Lat2* ^−/−^ mice are more susceptible to *C. albicans* infection than wild type (WT) mice. Dendritic cells (DCs) express LAB and we show that it is basally phosphorylated by the growth factor M-CSF or following engagement of Dectin-2, but not Dectin-1. Our data revealed a unique mechanism whereby LAB controls basal and fungal/pathogen-associated molecular patterns (PAMP)-induced nuclear β-catenin levels. This in turn is important for controlling fungal/PAMP-induced cytokine production in DCs. *C. albicans-* and LPS-induced IL-12 and IL-23 production was blunted in *Lat2^−/−^* DCs. Accordingly, *Lat2^−/−^* DCs directed reduced Th1 polarization *in vitro* and *Lat2* ^−/−^ mice displayed reduced Natural Killer (NK) and T cell-mediated IFN-γ production *in vivo/ex vivo*. Thus our data define a novel link between LAB and β-catenin nuclear accumulation in DCs that facilitates IFN-γ responses during anti-fungal immunity. In addition, these findings are likely to be relevant to other infectious diseases that require IL-12 family cytokines and an IFN-γ response for pathogen clearance.

## Author Summary

Fungal infections are a major healthcare problem and the incidence of fungal infections has increased significantly in recent years. Mortality rates are high even with treatment, highlighting the need for a better understanding of anti-fungal immunity in order to develop improved therapies. Adaptive T-helper 1 and T-helper 17 (Th1 and Th17) responses are important mediators of anti-fungal immunity. Dendritic cells express Dectin-1, Dectin-2 and Toll-like receptors, which interact with fungal pathogens to induce these adaptive immune responses. Here we identify LAB as an important facilitator of IFN-γ production by regulating β-catenin activation. Susceptibility to fungal infections is increased in the absence of LAB, in association with reduced IFN-γ production. β-catenin activation in dendritic cells inhibits the IL-12 production required for IFN-γ production. Thus targeting β-catenin therapeutically could help to promote efficient IFN-γ production in patients suffering from fungal infections. These findings are important for fungal infections and potentially for other diseases where IFN-γ production is important for disease outcome.

## Introduction

Fungal infections with pathogens such as *Candida albicans* are a significant health risk for immunocompromised individuals [1]. There is a high degree of mortality in these cases even with treatment, highlighting the need for a better understanding of the immune response involved in controlling fungal infections in order to develop improved treatments [2], [3]. Responses to fungal infections involve both innate and adaptive immunity [4]. The host response relies on the recognition, ingestion and elimination of *C. albicans* by phagocytic cells. During fungal infections, various pro-inflammatory cytokines such as TNF, IL-12p70, IL-23 and IL-6, produced by the activated leukocytes, result in the promotion of a sustained Th1 and Th17 response [5], [6], [7]. The requirement for these cytokines and pathways has been demonstrated by increased susceptibility of several knockout mice to *C. albicans* infections. For example, mice deficient in genes associated with Th1 responses such as *Il12a*, *Ifng* or *Ifngr1* are more susceptible to systemic *C. albicans* infection [8], [9]. In addition, *Il18* ^−/−^ mice display enhanced susceptibility to disseminated *C. albicans* due to their inability to produce sufficient IFN-γ [10]. More recently, fungal responses have been shown to involve the Th17 pathway; *Il23a^−/−^*, *Il17ra^−/−^* and *Il17a^−/−^* mice are more susceptible to oral and/or systemic candidiasis [6], [11], [12]. Therefore, the level of inflammatory cytokine production in response to *C. albicans* infection is important in determining whether the host will eliminate or succumb to the infection.

Stimulation of host immune cells to produce pro-inflammatory cytokines occurs through the recognition of PAMPs by pathogen-recognition receptors (PRRs) [13]. Various PRRs such as the mannose receptor, TLR2/4, CR3, Dectin-1 and Dectin-2 are involved in fungal recognition and responses [14]. Dectin-1 and Dectin-2 are type II, C type lectin-like receptors expressed mainly on myeloid cells [15], [16]. Fungal cell walls are mostly composed of β-glucans, chitins and mannans. Dectin-1 recognizes β-glucans in the fungal cell wall while Dectin-2 binds mannans. Mice lacking either Dectin-1 (*Clec7a^−/−^*) or Dectin-2 (*Clec4n^−/−^*) exhibit increased susceptibility to fungal infections supporting the role of these proteins in fungal immunity [5], [6], [17], [18]. Furthermore, identification of a *CLEC7A* single nucleotide polymorphism in humans, which encodes a non-functional form of Dectin-1, confirms the role of Dectin-1 in anti-fungal responses, as carriers of this polymorphism are more susceptible to mucocutaneous infections with *C. albicans*, in part due to reduced IL-17 production [19], [20]. Consistent with these data, polymorphisms resulting in IL-17RA and IL-17F deficiency were recently identified in some patients with chronic mucocutaneous *candidiasis* [21].

A wide variety of immunoreceptor tyrosine-based activation motif (ITAM)-coupled receptors are centrally involved in mediating the inflammatory response. The Dectin-1 cytoplasmic domain includes an ITAM-like sequence (known as a hemiITAM, YxxxI/Lx_7_YxxL) while Dectin-2 couples to the signaling chain FcεRIγ, which signals via a canonical ITAM (YxxLx_7–12_YxxL) [13]. Engagement of ITAM-associated receptors such as Dectin-2 (FcεRIγ), or hemiITAM-containing receptors such as Dectin-1, results in phosphorylation of the tyrosines within ITAMs or hemiITAMs. Src homology domain-2 containing protein tyrosine kinases of the Syk/Zap70 family are then recruited to the ITAM/hemiITAM and activated, resulting in CARD9- and MAPK-dependent pro-inflammatory cytokine production [4]. Surprisingly however, the biochemical adaptor(s) involved in coupling Dectin-1 or Dectin-2 proximal phosphorylation to downstream effectors have yet to be identified and despite the established importance of these receptors, little is known about the regulation of their signaling. Two closely related transmembrane adaptor proteins, the Linker for Activation of T cells (LAT/*Lat1*) and Linker for Activation of B cells/Non-T cell Activation Linker/Linker for Activation of T cells family member 2 (LAB/NTAL/*Lat2*, hereafter referred to as LAB) facilitate signaling downstream of various ITAM-coupled receptors [22]. These adaptor proteins activate signaling pathways such as the MAPK cascade and regulate production of cytokines including IL-12p40 [23]. As a consequence we hypothesized that LAT and/or LAB would mediate or regulate cytokine production during fungal responses downstream of Dectin-1 and/or Dectin-2 due to the similarities in hemiITAM and canonical ITAM signaling.

Here we demonstrate that LAB is involved in anti-fungal immunity. Mice deficient in LAB (*Lat2^−/−^* mice) are more susceptible to systemic *C. albicans* infection than WT mice. However, *Lat2^−/−^* neutrophil cytokine production is not impaired suggesting a defect in another cell type. We show that LAB but not LAT, is expressed by DCs and that both the M-CSF/DAP12 and mannan-Dectin-2/FcεRIγ pathways promote LAB phosphorylation. Conversely, the β-glucan-Dectin-1 or TLR pathways do not. We also define a novel role for LAB in suppressing β-catenin nuclear translocation, which in turn permits efficient IL-12 production from bone marrow-derived DCs (BMDCs) stimulated with a range of PAMPs. Furthermore, through this novel mechanism LAB promotes NK and T cell-mediated IFN-γ production, which is deficient *in vivo* during *C. albicans* infection of *Lat2^−/−^* mice. Thus LAB provides a molecular bridge between β-catenin activation and the cytokine production required for fungal clearance during systemic infection.

## Results

### *Lat2^−/−^* mice display increased susceptibility to challenge with *C. albicans*

**Figure 1:**
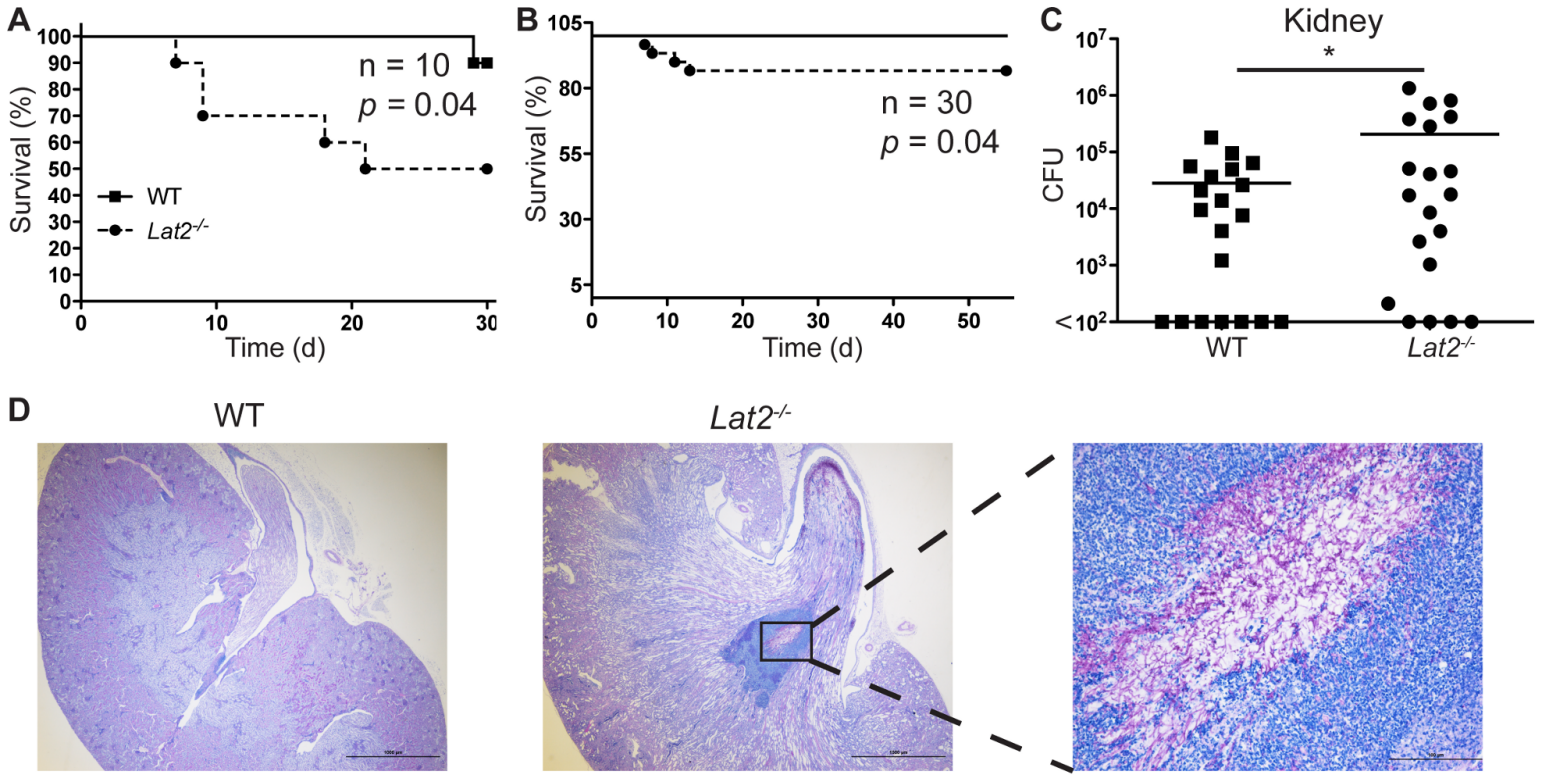
*Lat2^−/−^* mice display increased susceptibility to *C. albicans*. Survival curves of WT (filled squares) and *Lat2^−/−^* mice (filled circles) infected intravenously with (A) 1.5×10^5^ CFU or (B) 5×10^4^ CFU *C. albicans* SC5314. (A) Graph is representative of 3 independent experiments. *p = 0.04* (log-rank test), *n = 10*. (B) Graph is the cumulative result of 3 independent experiments. *p = 0.04* (log-rank test), *n = 30*. (C) CFU in the kidneys at 9 days after infection with 1.5×10^5^ CFU *C. albicans*. Graph is the cumulative result of 4 independent experiments. *p<0.05 (Student's *t* test on transformed data). Each symbol represents an individual mouse. (D) Fungal growth in a representative WT (left panel (2× magnification)) or *Lat2^−/−^* (middle panel (2x) and enlargement of boxed area, right panel (20x)) kidney at time of death (*Lat2^−/−^*) or 55 days (WT) after i.v. infection with 5×10^4^ CFU *C. albicans*. Kidney sections were stained with Periodic Acid Schiff.

As LAB has previously been shown to mediate/regulate signaling and/or cytokine responses downstream of ITAM-coupled receptors and TLRs [23], [24], we hypothesized that LAB would play a role in anti-fungal immunity. To investigate this possibility, we systemically infected WT and *Lat2^−/−^* mice with *C. albicans*. *Lat2^−/−^* mice displayed increased susceptibility to high (1.5×10^5^) and low (5×10^4^) dose *C. albicans* infection compared to WT mice (Fig. 1A–B). The reduced survival was paralleled by increased fungal burden in the kidneys of *Lat2^−/−^* mice, nine days after infection (i.v.) with *C. albicans* (Fig. 1C). The kidney depicted from a *Lat2^−/−^* mouse that succumbed to infection displays a marked proliferation of fungal hyphae within the pelvis, which was surrounded by neutrophilic inflammation, consistent with an inability to clear the infection (Fig. 1D). Together, these data demonstrate that LAB is important for the host response to *C. albicans* infection.

### *Lat2^−/−^* neutrophil cytokine production is not impaired

**Figure 2:**
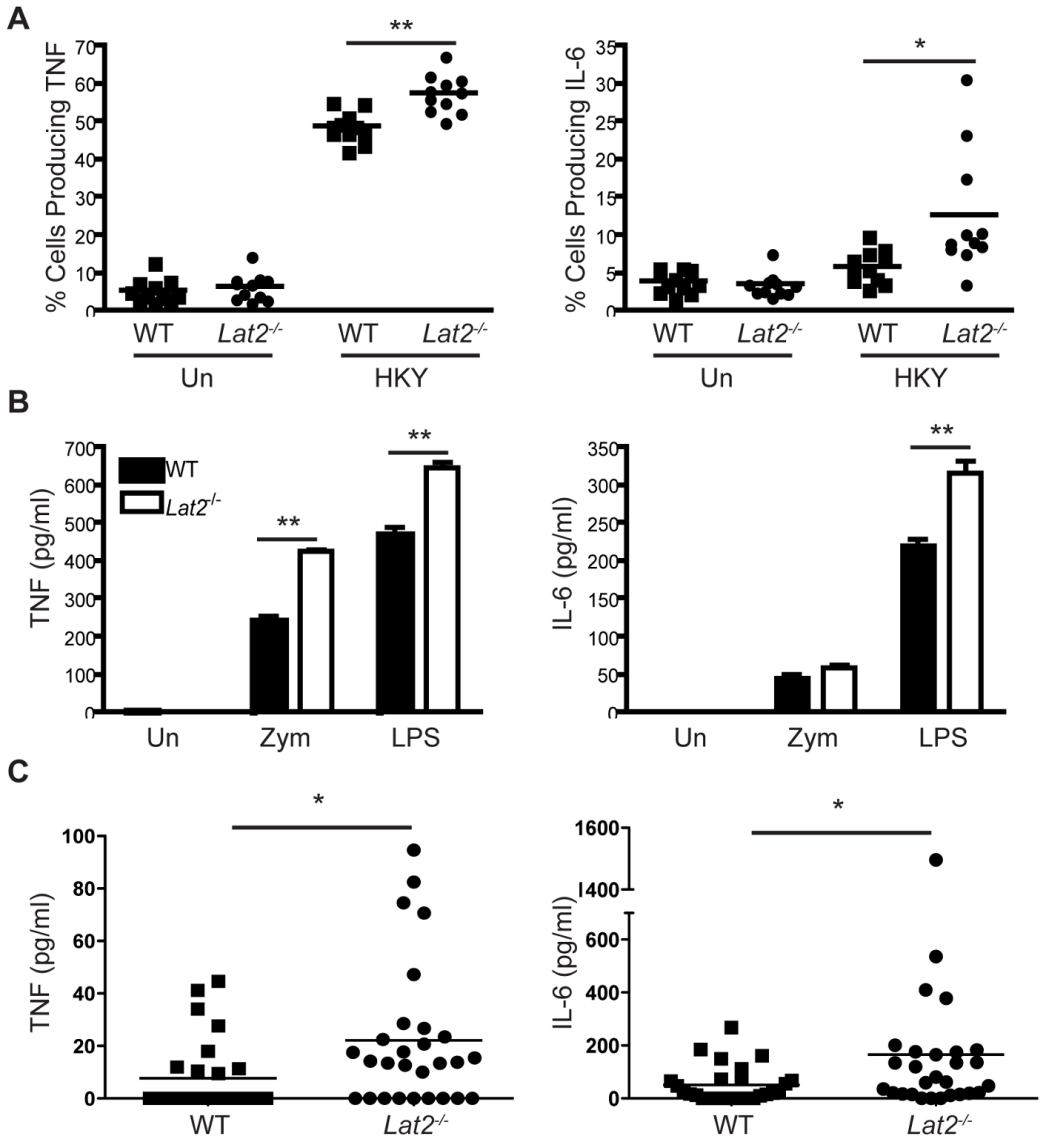
*Lat2^−/−^* neutrophil cytokine responses are not impaired. (A) Peritoneal neutrophils (from 4 h intraperitoneal infection with 1×10^5^ CFU *C. albicans*) were re-stimulated with/without heat-killed *C. albicans* yeast. TNF and IL-6 levels were analyzed by flow cytometry. Graph is the cumulative result of 2 independent experiments. Each symbol represents an individual mouse. *p<0.05 **p<0.005 (1-way ANOVA, Bonferroni's post-test) (B) Purified bone marrow neutrophils were stimulated with 10 µg/ml zymosan or 100 ng/ml LPS. TNF and IL-6 levels in the supernatants from three replicates were measured after 24 h. Data are representative of 2 independent experiments. **p<0.005 (1-way ANOVA, Bonferroni's post-test) (C) TNF and IL-6 levels were measured in the serum of WT (filled squares) and *Lat2^−/−^* mice (filled circles) at time of death or 30 days after i.v. infection with 1.5×10^5^ CFU *C. albicans* SC5314. Graph is the cumulative result of 3 independent experiments. Each symbol represents an individual mouse. *p<0.05 (TNF - Mann Whitney test; IL-6 - Student's *t* test on transformed data).

Neutrophil function is important for anti-fungal immunity. They produce cytokines such as TNF and IL-6, both of which play a role in determining susceptibility to fungal infections [25], [26]. As LAB is expressed in neutrophils [24], we examined the effect of LAB deficiency on *C. albicans*-recruited neutrophil cytokine production following restimulation with heat-killed *C. albicans* yeast (HKY). TNF and IL-6 production from *C. albicans*-recruited neutrophils was not impaired in *Lat2^−/−^* cells, in fact they were enhanced (Fig. 2A), similar to the findings of Tessarz *et al* [24]. Consistent with these data, we observed enhanced TNF and IL-6 production from bone marrow purified neutrophils following stimulation with zymosan and LPS (Fig. 2B). In addition, serum TNF and IL-6 levels were enhanced in *Lat2^−/−^* mice systemically infected with *C. albicans* (Fig. 2C). These data indicate that neutrophil cytokine production is not impaired in *Lat2^−/−^* mice suggesting that LAB plays additional roles in other cell types during systemic *C. albicans* infection.

### LAB is downstream of zymosan and *C. albicans* signaling

**Figure 3:**
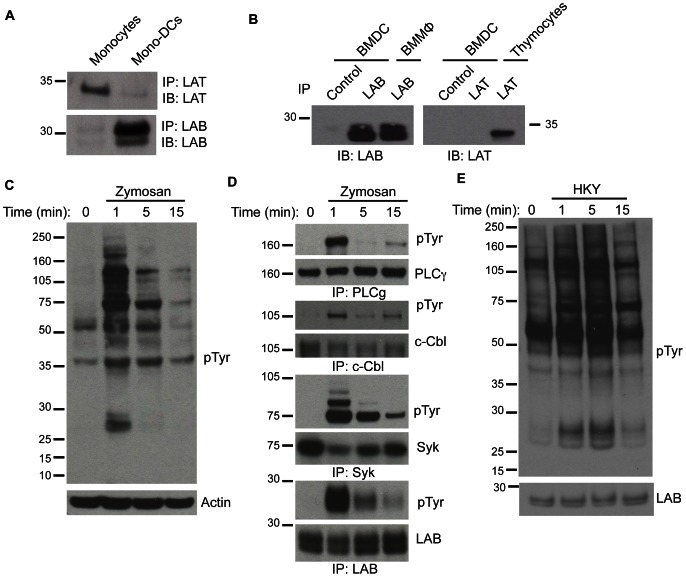
LAB expression in DCs and involvement in zymosan and *C. albicans* signaling. (A & B) Human (A) and mouse (B) cells were immunoprecipitated with anti-LAT and anti-LAB, resolved via SDS-PAGE and immunoblotted with anti-LAT and anti-LAB. (C–E) BMDCs were stimulated with 1 mg/ml zymosan (C–D) or 1×10^7^ heat-killed *C. albicans* yeast (E) for the indicated times and (C) whole cell lysates (WCL) were resolved via SDS-PAGE and immunoblotted with anti-phosphotyrosine and reprobed with anti-Actin. (D) Cells were immunoprecipitated with anti-PLCγ2, anti-c-Cbl, anti-Syk or anti-LAB and immunoblotted with anti-phosphotyrosine, anti-PLCγ2, anti-c-Cbl, anti-Syk or anti-LAB. (E) WCL were immunoblotted with anti-phosphotyrosine and anti-LAB. Data are representative of 2–3 independent experiments.

In addition to neutrophils, DCs are important for anti-fungal immunity. In previous studies, we have shown that there is a switch from LAT to LAB expression as monocytes differentiate into macrophages and that LAB is involved in regulating ITAM-mediated signals in macrophages [23]. To extend these studies to DCs we examined the expression of LAB in human monocytes and monocyte-derived DCs and murine GM-CSF-cultured BMDCs. Similar to macrophages, LAB is expressed in DCs while LAT expression is lost (Fig. 3A–B). To determine whether LAB is involved in fungal-induced signaling, we stimulated BMDCs with the yeast cell wall extract zymosan and found that many proteins were tyrosine phosphorylated following stimulation (Fig. 3C). Consistent with previous findings [5], [27] we found that Syk and PLCγ2 were prominently phosphorylated following zymosan stimulation (Fig. 3D). This response was also associated with the phosphorylation of LAB and one of its binding partners, c-Cbl [23] (Fig. 3D). Phosphorylation of these proteins was unaffected by the absence of LAB (Fig. S1). Accordingly, we found that activation of the Erk and NFκB pathways were unaffected by the absence of LAB (Fig. S1). Similar to our findings with zymosan, we also observed that many proteins, including LAB, were phosphorylated following stimulation with heat-killed *C. albicans* yeast (HKY) (Fig. 3E). The zymosan- and *C. albicans*-induced phosphorylation of LAB in DCs suggests that this adaptor protein may be important in regulating downstream pathways in DCs that are instrumental in combating fungal infections.

### Impaired cytokine responses in *Lat2^−/−^* DCs

**Figure 4:**
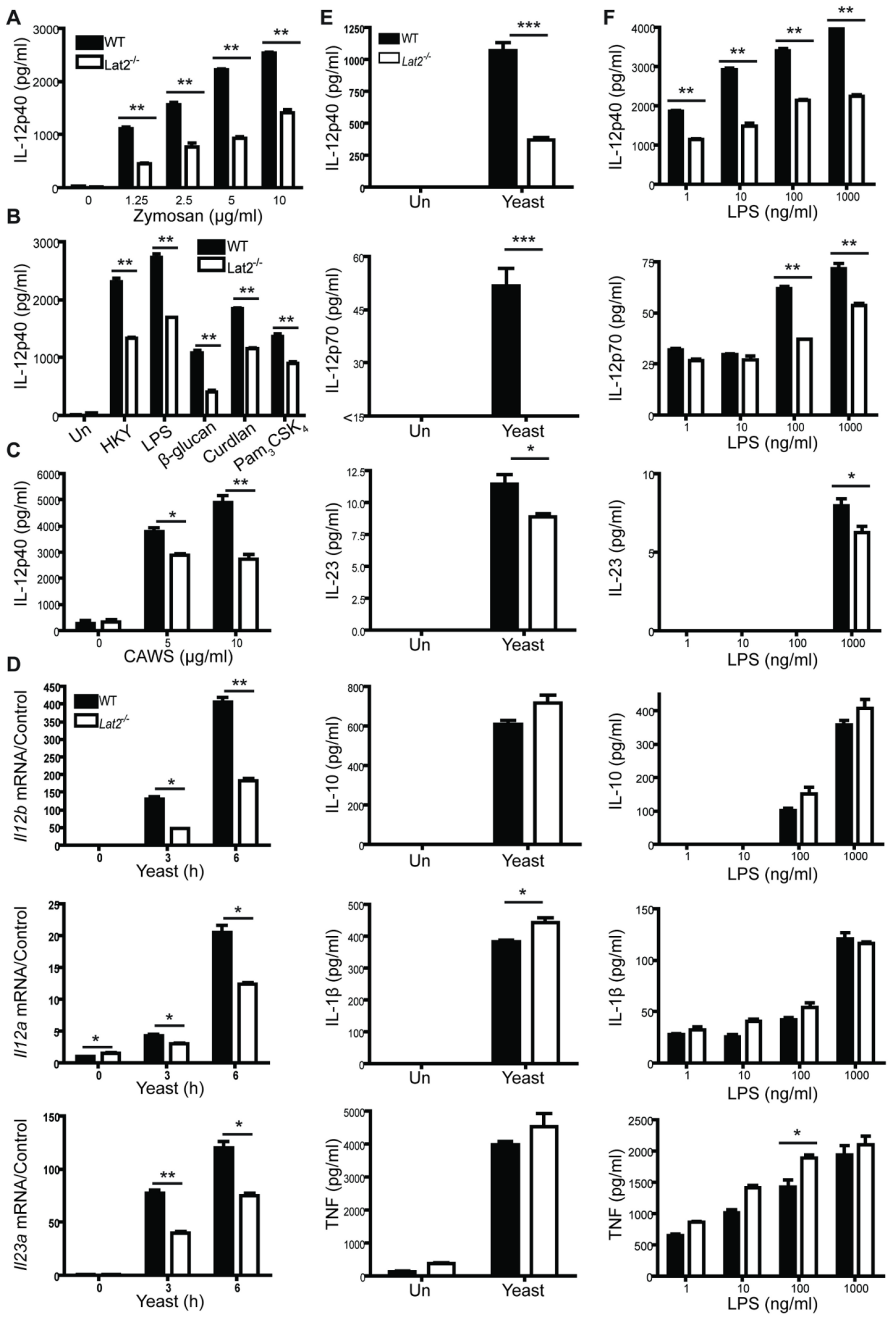
LAB facilitates PAMP/fungal-induced production of IL-12 and IL-23 by BMDCs. (A–C) BMDCs from WT and *Lat2* ^−/−^ mice were stimulated with (A) zymosan or (B) 1×10^5^ HKY, 100 ng/ml LPS, 5 µg/ml particulate β–glucan, 10 µg/ml Curdlan, 20 ng/ml Pam_3_CSK_4_ or (C) 5–10 µg/ml CAWS (mannans). Cytokine levels in the supernatants were measured after 24 h incubation (B) or 48 h incubation (C). (D) BMDCs from WT and *Lat2* ^−/−^ mice were stimulated for the indicated times with 1×10^5^ live *C. albicans* yeast. RNA was isolated, cDNA was prepared and *Il12b*, *Il12a* and *Il23a* mRNA transcripts were detected by real-time qPCR. mRNA levels were normalized to *Hprt1*. Black bars represent WT, white bars represent *Lat2* ^−/−^. (E) BMDCs from WT and *Lat2* ^−/−^ mice were stimulated for the indicated times with live *C. albicans* yeast. Fungizone was added 2 h later and cytokine levels in the supernatants were measured after 24 h incubation. (F) BMDCs from WT and *Lat2* ^−/−^ mice were stimulated with 1–1000 ng/ml LPS. Cytokine levels in the supernatants were measured after 24 h incubation. For all graphical data, results are presented as means +/− s.e.m. of three replicates and data are representative of 2–4 independent experiments. *p<0.05 **p<0.005 ***p<0.0005 (1-way ANOVA, Bonferroni's post-test or Student's *t* test).

Given that LAB is phosphorylated following zymosan stimulation in DCs, we next assessed the ability of LAB to regulate zymosan-induced cytokine responses in DCs. We found that zymosan-induced IL-12p40 production from BMDCs is partially dependent on LAB (Fig. 4A). As zymosan is a complex ligand that engages multiple receptor systems, including Dectin-1, Dectin-2, TLR2 and others, we examined the effect of LAB on various fungal and TLR ligands. WT and *Lat2^−/−^* BMDCs were stimulated with heat-killed *C. albicans* yeast, LPS, particulate β–glucan (Sigma), curdlan, Pam_3_CSK_4_ and CAWS (mannans). IL-12p40 production was significantly reduced in *Lat2^−/−^* BMDCs following stimulation with each of these ligands suggesting that LAB plays an important role in IL12-p40 production (Fig. 4B–C). To underline the importance of these findings to fungal infection, we next stimulated BMDCs with the yeast form of live *C. albicans*. These results showed that induction of *Il12b*, *Il12a* and *Il23a* mRNA were significantly impaired in *Lat2^−/−^* cells compared to WT controls (Fig. 4D). Subsequently, after a twenty-four hour stimulation of BMDCs with live *C. albicans*, cytokine levels in the supernatants were measured. These data showed that production of IL-12p40 and IL-12p70 were both greatly reduced, and IL-23 levels were also partially suppressed, in *Lat2^−/−^* BMDCs following stimulation with live yeast. However, IL-10, IL-1β and TNF levels were not impaired by the absence of LAB (Fig. 4E). We next stimulated BMDCs with increasing doses of LPS. Consistent with our findings with *C. albicans* we demonstrated that LAB plays a significant role in mediating the production of IL-12 family cytokines downstream of LPS, while production of IL-10, IL-1β and TNF levels were not impaired by the absence of LAB (Fig. 4F). These data indicate that LAB plays an important and selective role in facilitating fungal/PAMP-induced IL-12 family cytokine production.

### LAB is required to induce an efficient Th1 response *in vitro*

**Figure 5:**
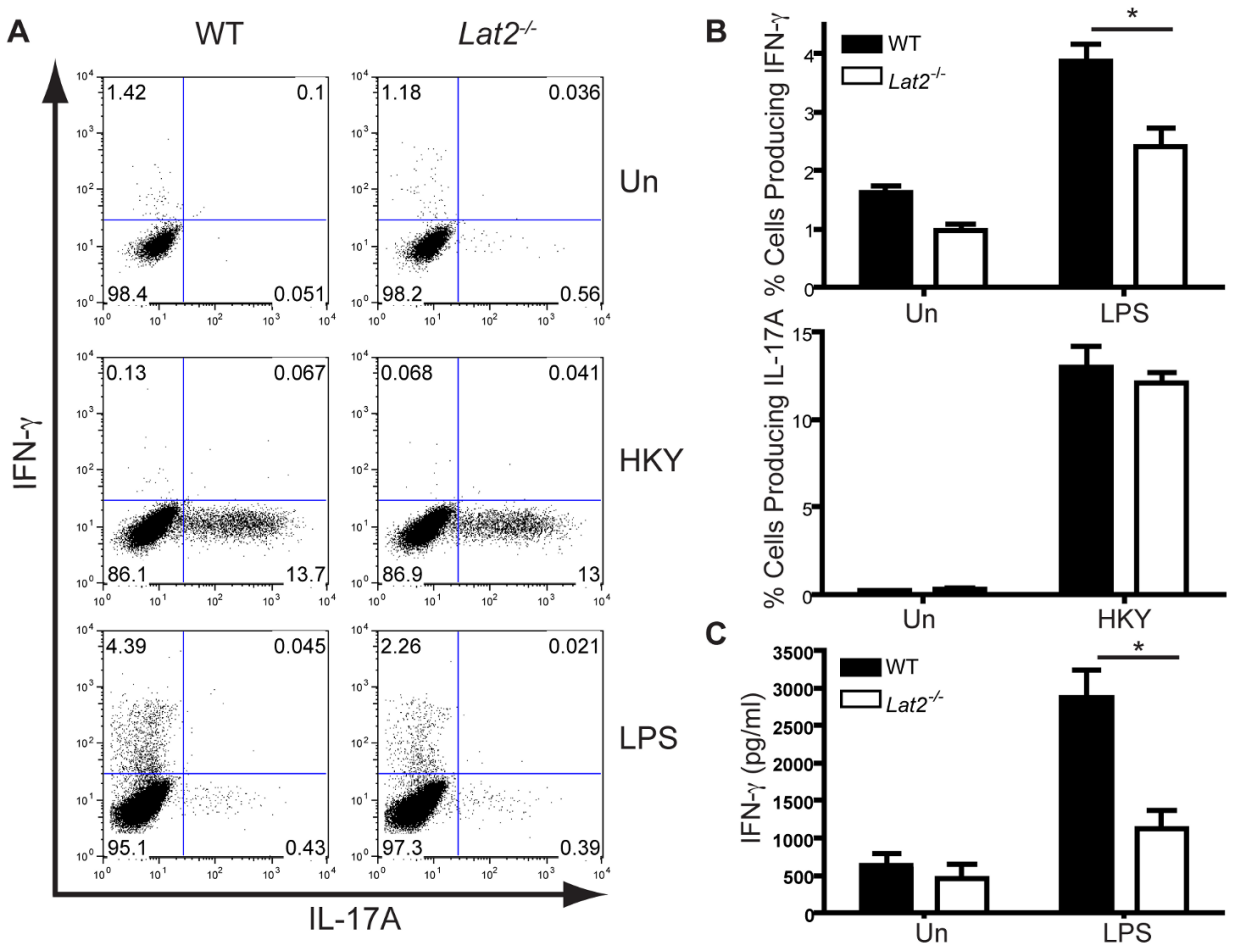
LAB is required for efficient Th1 responses. (A–C) Purified wild-type naïve CD4^+^ T cells were stimulated with anti-CD3 and anti-CD28 for 4 days in the presence of conditioned medium from BMDCs cultured with 1×10^5^ heat-killed *C. albicans* yeast or 1 µg/ml LPS. (A–B) The cells were restimulated with PMA and Ionomycin and IFN-γ and IL-17A levels were analyzed by flow cytometry. Flow plots are representative of three replicates. (B) Graphs display mean +/− s.e.m. % cells expressing IFN-γ or IL-17A from three replicates analyzed by flow cytometry. Black bars represent WT, white bars represent *Lat2* ^−/−^. (C) IFN-γ levels in the supernatants from three replicates were measured after 4 days. Data are representative of 3 independent experiments. *p<0.05 (1-way ANOVA, Bonferroni's post-test).

*C. albicans* contains ligands for many receptors (including Dectin-1, Dectin-2 and TLRs 2 and 4) and systemic infection with *C. albicans* involves both Th1 and Th17 responses [5]. *In vitro* however, the T cell response to *C. albicans* is dominated by Th17 polarization [6]. In order to assess the capacity of *Lat2^−/−^* DC to direct both Th17 and Th1 responses in the context of reduced IL-12 production, heat-killed *C. albicans* and the alternate TLR4 selective ligand, LPS, were used respectively. Supernatants from WT and *Lat2^−/−^* BMDCs cultured in the presence of heat-killed *C. albicans* yeast or LPS were added to naïve WT CD4^+^ T cells stimulated with anti-CD3 and anti-CD28. IFN-γ and IL-17A production were measured by flow cytometry and ELISA following 4 days of culture. As expected, conditioned media from BMDCs stimulated with heat-killed yeast induced a robust Th17 response while stimulation with LPS induced a Th1 response (Fig. 5A-B). HKY-stimulated WT and *Lat2^−/−^* BMDCs induced comparable Th17 responses (Fig. 5A–B), likely due to the production of similar levels of IL-1β and only a minimal defect in IL-23 production from *Lat2^−/−^* BMDCs. Interestingly, the proportion of IFN-γ-secreting CD4^+^ T cells arising from LPS stimulated *Lat2^−/−^* BMDCs was significantly reduced compared to WT BMDCs (Fig. 5A–C), which could be attributed to the significant reduction in IL-12p70 production from *Lat2^−/−^* BMDCs (Fig. 4E–F). These data indicate that LAB-facilitated cytokine production is important for inducing Th1 responses but not Th17 responses *in vitro*. As both Th1 and Th17 responses are important for *in vivo* protection against *C. albicans* infections [6], [9], our data suggests that defective Th1 responses in *Lat2^−/−^* mice may be responsible for the increased susceptibility of these mice to *C. albicans* infection.

### Signaling through Dectin-2 promotes LAB phosphorylation

**Figure 6:**
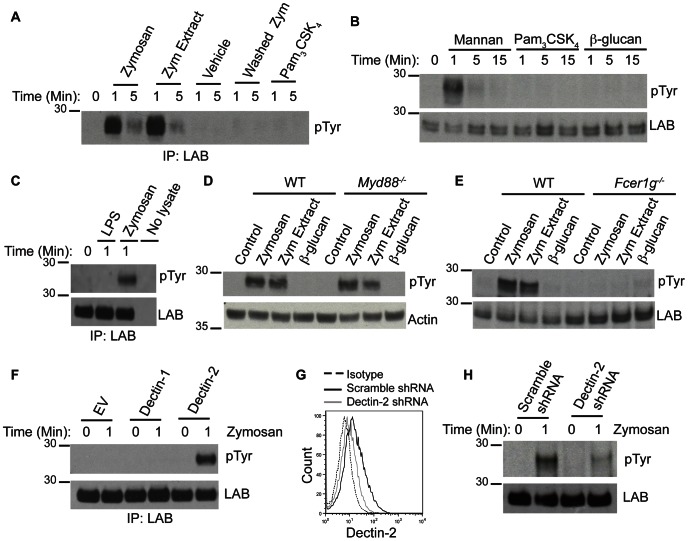
Mannan-Dectin-2 signaling stimulates LAB phosphorylation. (A) BMDCs were stimulated with 1 mg/ml zymosan, zymosan extract, vehicle control, washed zymosan or Pam_3_CSK_4_ for the indicated times and cells were immunoprecipitated with anti-LAB and immunoblotted with anti-phosphotyrosine. (B) BMDCs were stimulated with 1 mg/ml mannan, particulate β-glucan or Pam_3_CSK_4_ for the indicated times and WCL were immunoblotted with anti-phosphotyrosine and anti-LAB. (C) BMDCs were stimulated with 10 µg/ml LPS or 1 mg/ml zymosan for the indicated times and WCL were immunoprecipitated with anti-LAB and immunoblotted with anti-phosphotyrosine and anti-LAB. (D & E) BMDCs from WT, *Myd88^−/−^* (D) and *Fcer1g^−/−^* (E) mice were stimulated for 1 min with 1 mg/ml zymosan, zymosan extract or particulate β-glucan. WCL were immunoblotted with anti-phosphotyrosine and anti-Actin or anti-LAB. (F) RAW-264 cell lines expressing Empty Vector (EV), Dectin-1 or Dectin-2 were stimulated with 1 mg/ml zymosan. Cells were immunoprecipitated with anti-LAB and immunoblotted with anti-LAB and anti-phosphotyrosine. (G–H) BMDCs were infected with scrambled shRNA control or Dectin-2 shRNA. (G) BMDCs were stained for surface expression of Dectin-2 and analyzed by flow cytometry. (H) BMDCs were stimulated with 1 mg/ml zymosan and WCL were immunoblotted with anti-phosphotyrosine and anti-LAB.

As LAB facilitates fungal/PAMP-induced cytokine production in DCs and subsequent Th1 polarization, we next sought to determine which signaling pathways were involved. As LAB phosphorylation is a documented feature of ITAM-coupled receptor signaling [23], [28], we hypothesized that β-glucans signaling through the hemiITAM-containing Dectin-1 or mannans signaling through the ITAM-coupled Dectin-2 would induce LAB phosphorylation. After resting the cells to remove basal phosphorylation, we found, surprisingly, that stimulation of DCs with washed zymosan particles (insoluble components of zymosan such as the β-glucans) or purified particulate β-glucans does not lead to LAB phosphorylation while stimulation with zymosan extract (soluble components of zymosan such as the mannans) or purified mannans caused rapid and robust LAB phosphorylation (Fig. 6A–B). LAB phosphorylation was not observed in response to TLR2/4 ligands Pam_3_CSK_4_ or LPS (Fig. 6A–C). These data suggest that LAB may be phosphorylated downstream of the mannan-Dectin-2/FcεRIγ pathway rather than via Dectin-1 or the TLR pathways. To further address this possibility, we examined LAB phosphorylation in DCs lacking *Myd88* or *Fcer1g*. Consistent with a role for Dectin-2, LAB phosphorylation was intact in the absence of MyD88 (Fig. 6D) and ablated in *Fcer1g^−/−^* cells (Fig. 6E). We next stimulated RAW-264 macrophage cell lines, engineered to express either Dectin-1 or Dectin-2 [29], with zymosan and found that LAB was only phosphorylated in cells expressing Dectin-2 (Fig. 6F). This was in spite of high zymosan recognition by Dectin-1 over-expressing RAW-264 macrophages and their potential to signal via TLRs [29], [30]. To confirm this selective role for Dectin-2, we conducted Dectin-2 specific shRNA-knockdown experiments (Fig. 6G–H). We found that LAB phosphorylation was attenuated in DCs with partial Dectin-2 knockdown as a result of infection with shRNA against Dectin-2 compared to BMDCs infected with a scrambled shRNA control (Fig. 6H). Taken together, these data clearly demonstrate that zymosan stimulates LAB phosphorylation through a mannan-driven Dectin-2-FcεRIγ signaling pathway and not through the β-glucan-Dectin-1 or TLR pathways. The phosphorylation of LAB downstream of Dectin-2 could potentially explain the reduced IL-12p40 in response to *C. albicans*, zymosan and mannans. However, the reduced IL-12p40 in response to β-glucan, LPS or Pam_3_CSK_4_ implies the involvement of another LAB activating pathway.

### Signaling through M-CSF/DAP12 promotes LAB phosphorylation

**Figure 7:**
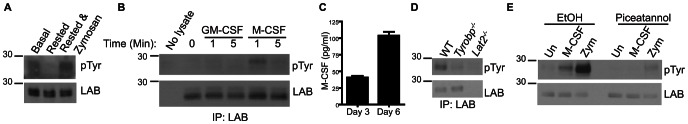
MCSF/DAP12 signaling stimulates LAB phosphorylation. (A) BMDCs from WT mice were lysed immediately without resting or rested for 30 min in serum free media +/− stimulation with 1 mg/ml zymosan for 1 min. WCL were immunoblotted with anti-LAB and anti-phosphotyrosine. (B) BMDCs from WT mice were stimulated for the indicated times with 100 ng/ml GM-CSF and M-CSF. WCL were immunoprecipitated with anti-LAB and immunoblotted with anti-phosphotyrosine and anti-LAB. (C) M-CSF levels in supernatants from WT BMDC cultures at Days 3 and 6 were measured. (D) BMDCs from WT, *Tyrobp^−/−^* and *Lat2^−/−^* mice were lysed immediately without resting. WCL were immunoprecipitated with anti-LAB and immunoblotted with anti-phosphotyrosine and anti-LAB. (E) WT BMDCs were rested for 30 min, treated with 30 µg/ml Piceatannol or EtOH for 30 min prior to stimulation with 100 ng/ml M-CSF or 1 mg/ml zymosan for 1 min. WCL were immunoblotted with anti-phosphotyrosine and anti-LAB. Data are representative of 2 independent experiments.

Given the broad effect of LAB deletion, we hypothesized that LAB may exert its effect prior to stimulation. Therefore, we next examined whether LAB phosphorylation occurs in DC during their expansion *in vitro*. For this experiment we compared WT BMDCs harvested directly from culture to those rested in cytokine/serum free conditions for 30 min with or without subsequent stimulation with zymosan for 1 min (Fig. 7A). These data demonstrate readily appreciable basal phosphorylation of LAB in WT BMDCs during culture (Fig. 7A). LAB phosphorylation in response to SCF [31] has been described previously, suggesting that a growth factor may be responsible for this basal LAB phosphorylation. Therefore, we stimulated BMDCs with GM-CSF and M-CSF. Interestingly, M-CSF induced LAB phosphorylation (Fig. 7B) and examination of the BMDC cultures demonstrated that while M-CSF is not added to these cultures, the cells are producing appreciable levels of M-CSF (Fig. 7C) during culture. Crosstalk between M-CSF and the ITAM-containing signaling chain, DAP12 has recently been described [32]. Thus, we tested whether basal LAB phosphorylation in BMDC cultures was DAP12-dependent. Basal levels of LAB phosphorylation were almost completely abolished in the absence of DAP12 (Fig. 7D). We then sought to determine whether M-CSF-induced LAB phosphorylation was dependent on Syk. Pre-treatment of WT cells with the Syk inhibitor, Piceatannol, inhibited M-CSF- and zymosan-induced LAB phosphorylation (Fig. 7E). These data indicate that LAB is phosphorylated through an M-CSF/DAP12-Syk pathway during culture. This basal M-CSF/DAP12 pathway may control IL-12p40 production in response to all of the PAMPs in this study.

### LAB facilitates fungal-induced cytokine production by controlling β-catenin activation

**Figure 8:**
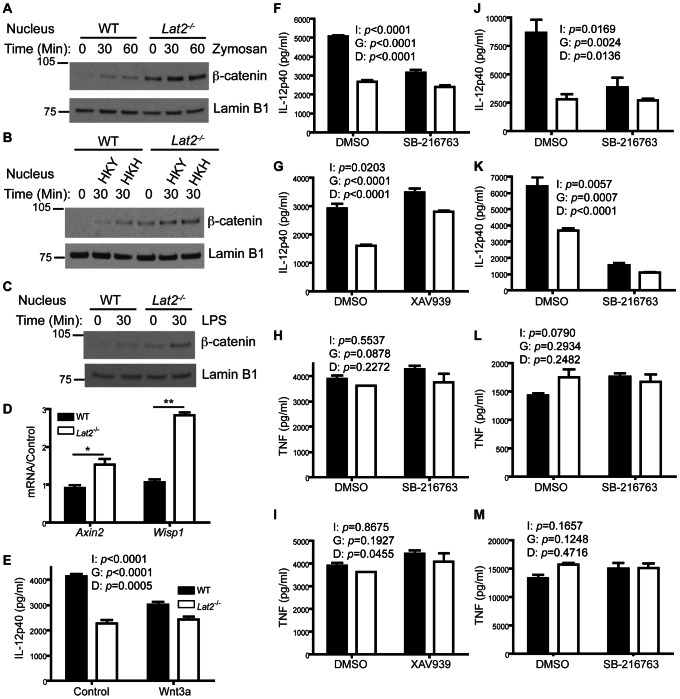
LAB facilitates fungal-induced cytokine production by controlling β-catenin activation. (A–C) BMDCs from WT and *Lat2* ^−/−^ mice were stimulated for the indicated times with (A) 1 mg/ml zymosan, (B) 1×10^7^ heat-killed *C. albicans* yeast or hyphae or (C) 10 ng/ml LPS. Nuclear fractions were immunoblotted with anti-β-catenin and reprobed with anti-Lamin B1. (D) RNA was isolated from WT and *Lat2* ^−/−^ BMDCs, cDNA was prepared and *Axin2* and *Wisp1* mRNA transcripts were detected by real-time qPCR. mRNA levels were normalized to *Hprt1*. Black bars represent WT, white bars represent *Lat2* ^−/−^. *p<0.05 **p<0.005 (1-way ANOVA, Bonferroni's post-test) (E) BMDCs from WT and *Lat2* ^−/−^ mice were treated with Vehicle Control or 100 ng/ml Wnt3a for 24 h prior to stimulation with heat-killed *C. albicans* yeast. Cytokine levels in the supernatants were measured after 24 h incubation. (F–I) BMDCs from WT and *Lat2* ^−/−^ mice were stimulated with heat-killed *C. albicans* yeast in the presence of DMSO, SB-216763 or XAV939. Cytokine levels in the supernatants were measured after 24 h incubation. (J–M) BMDCs from WT and *Lat2* ^−/−^ mice were stimulated with LPS (J&L), or WGP (K&M) in the presence of DMSO or SB-216763. Cytokine levels in the supernatants were measured after 24 h incubation (J–M). (E–M) A 2 way ANOVA was used for statistical analysis to examine for significant effects of the Genotype (G) and Drug (D) as well as an Interaction (I) between these two factors. For all graphical data, results are presented as means +/− s.e.m. of three replicates and data are representative of at least 2 independent experiments.

M-CSF is constitutively present in naïve mice *in vivo* and it is also induced during infection with *C. albicans* [33]. Moreover, M-CSF crosstalks with DAP12 to induce activation of β-catenin [32]. Importantly, β-catenin represses pro-inflammatory cytokine production in DCs [34], [35]. Therefore we examined nuclear β-catenin levels in WT and *Lat2^−/−^* BMDCs. Interestingly, *Lat2^−/−^* BMDCs showed increased β-catenin activation as evidenced by increased nuclear localization in response to zymosan, heat-killed yeast/hyphae (HKH) or LPS (Fig. 8A–C). Importantly, basal nuclear β-catenin levels are also increased in *Lat2^−/−^* BMDCs (Fig. 8A–C), and the physiological relevance of this was confirmed by the increased mRNA levels of the β-catenin target genes *Axin2* and *Wisp1* in non-stimulated cells (Fig. 8D). We next examined whether this increasedβ-catenin nuclear accumulation in *Lat2^−/−^* cells was responsible for the decreased cytokine production by these cells. In the absence of Wnt signaling β-catenin is phosphorylated by GSK-3β and targeted for proteasomal-mediated destruction [36]. We induced β-catenin stabilization by stimulating the Wnt pathway with the canonical ligand Wnt3a or with an inhibitor of GSK-3β (SB-216763). Following activation of β-catenin through Wnt3a or SB-216763, the difference in IL-12p40 production between WT and *Lat2^−/−^* cells was reduced (Fig. 8E–F) resulting in WT cells more closely resembling those deficient in LAB. In addition, we stimulated β-catenin degradation by stabilizing axin, a component of the destruction complex, with XAV939. This again resulted in alleviation of the difference in IL-12p40 production between WT and *Lat2^−/−^* cells with *Lat2^−/−^* cells more closely resembling WT cells (Fig. 8G). In all of the above cases, the two-way ANOVA indicates a clear interaction between the effect of the inhibitors/agonists and LAB-deficiency as predicted by the proposed role of β-catenin in LAB-facilitated IL-12p40 production. TNF levels were largely unaffected by treatment with inhibitors/activators of this pathway (Fig. 8H–I). As the basal and PAMP-induced β-catenin levels are different between WT and *Lat2^−/−^* BMDCs, we hypothesized that modulating β-catenin activation would normalize IL-12p40 production in response to a variety of PAMPs including those that do not directly stimulate LAB phosphorylation. Consistent with this hypothesis, activation of β-catenin through SB-216763 reduced the difference in IL-12p40 production between WT and *Lat2^−/−^* cells stimulated by LPS (TLR4) (Fig. 8J) or WGP (Dectin-1) (Fig. 8K). TNF levels were largely unaffected by treatment with SB-216763 in response to either LPS (Fig. 8L) or WGP (Fig. 8M). These data indicate that constitutive LAB phosphorylation maintains limited basal β-catenin nuclear accumulation, thus facilitating robust cytokine production, consistent with previous reports demonstrating that β-catenin represses pro-inflammatory cytokine production [34], [35].

### *Lat2^−/−^* mice display decreased DC and NK, T and NKT cell-mediated cytokine levels *in vivo*

**Figure 9:**
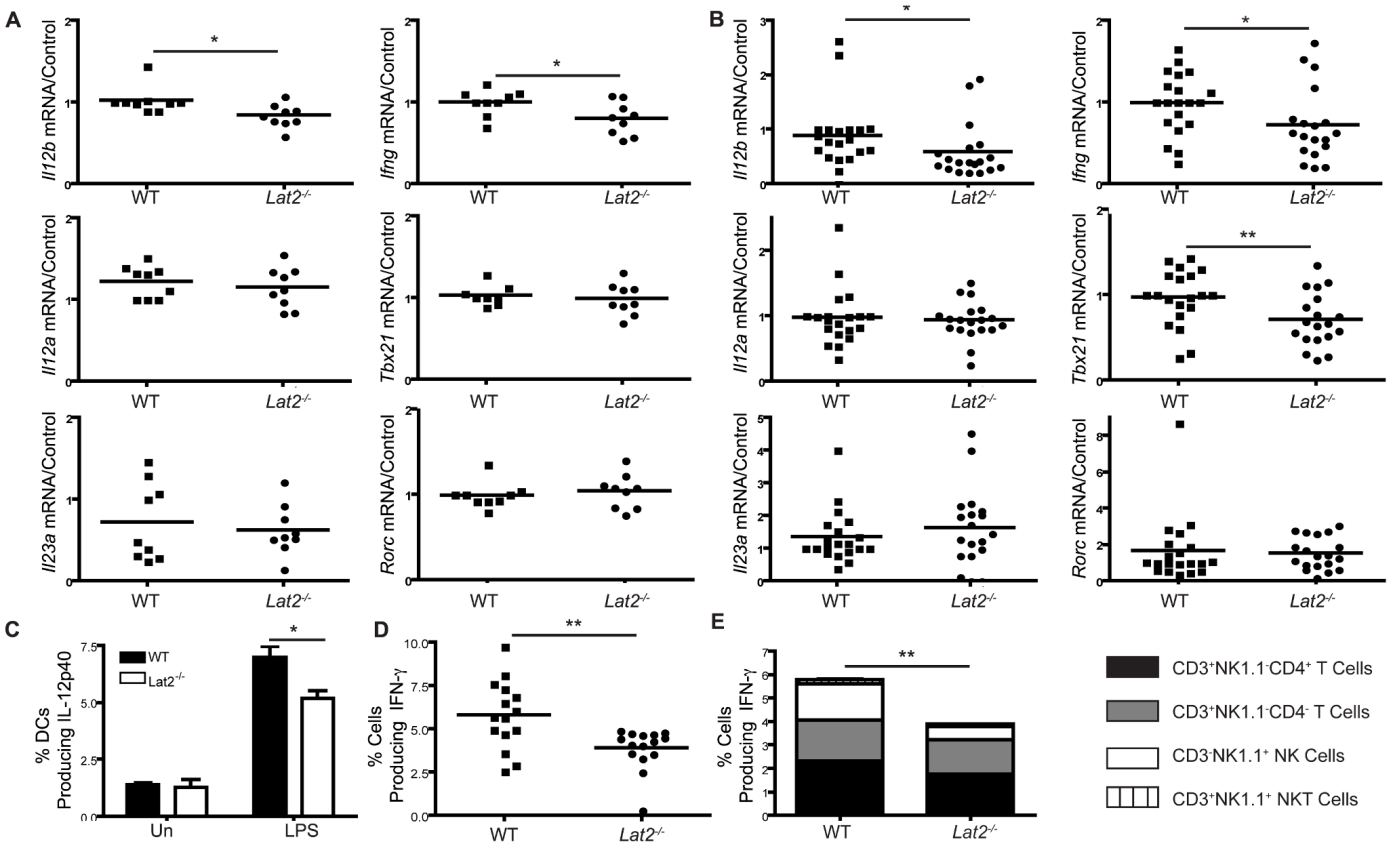
*Lat2^−/−^* mice display decreased DC and NK, T and NKT cell-mediated cytokine levels. (A–B) RNA was isolated from the spleens of (A) naïve WT and *Lat2* ^−/−^ mice and (B) WT and *Lat2* ^−/−^ mice 9 days after infection with 1.5×10^5^ CFU *C. albicans*, cDNA was prepared and mRNA transcripts were detected by real-time qPCR. mRNA levels were normalized to *Hprt1*. Graphs are the cumulative result of 3–4 independent experiments. *p<0.05 **p<0.005 (Student's *t* test). (B) *Il12b* data was transformed for analysis. (C) WT and *Lat2^−/−^* spleen cells were stimulated with LPS for 6 h. IL-12p40 levels in CD11c^+^MHCII^+^ DCs were analyzed by flow cytometry. Graph displays mean +/− s.e.m. % DCs expressing IL-12p40 from four mice analyzed by flow cytometry. Black bars represent WT, white bars represent *Lat2* ^−/−^. Data are representative of 2 independent experiments. *p<0.05 (1-way ANOVA, Bonferroni's post-test). (D–E) Peritoneal lavage cells from WT and *Lat2^−/−^* mice 72 h after intraperitoneal injection of *C. albicans* were re-stimulated with PMA/Ionomycin for 4 h. (D) Graph displays percentage of IFN-γ-producing cells. **p<0.005 (Student's *t* test). Each symbol represents an individual mouse. Graph is the cumulative result of 3 independent experiments. (E) Graph displays the mean percentage +/− s.e.m. of IFN-γ-producing CD3^+^NK1.1^−^CD4^+^, CD3^+^NK1.1^−^CD4^−^, CD3^−^NK1.1^+^ and CD3^+^NK1.1^+^ cells that combine to form total IFN-γ-producing cells in (D).

As *Lat2^−/−^* mice were more susceptible to systemic *C. albicans* infection and *Lat2^−/−^* DCs displayed impaired IL-12 production and subsequent Th1 responses *in vitro*, we wanted to determine whether these defective cytokine responses occurred *in vivo*. In correlation with the reduced IL-12p40 production *in vitro* we observed reduced *Il12b* mRNA levels in the spleens of both uninfected (Fig. 9A) and *C. albicans* infected *Lat2^−/−^* mice (Fig. 9B). Additionally, *Ifng* mRNA levels were reduced in both uninfected and *C. albicans* infected *Lat2^−/−^* mice and *Tbx21* mRNA levels were also reduced in the *C. albicans* infected *Lat2^−/−^* mice (Fig. 9A–B). In contrast *Il12a, Il23a* and *Rorc* mRNA levels were normal in the *Lat2^−/−^* mice and *Il17* mRNA levels were undetected. Consistent with reduced RNA levels of *Il12b*, we also observed reduced IL-12p40 in *Lat2^−/−^* splenic DCs following stimulation with LPS (Fig. 9C and Fig. S2). In order to further examine whether *Lat2^−/−^* mice displayed reduced IFN-γ responses, we injected WT and *Lat2^−/−^* mice intraperitoneally with *C. albicans* and collected cells by peritoneal lavage 72 h post injection. Following restimulation with PMA/Ionomycin there were reduced percentages of IFN-γ producing cells in *Lat2^−/−^* lavages (Fig. 9D and Fig. S2). The IFN-γ producing cells consisted of CD3^+^NK1.1^−^CD4^+^ T cells, CD3^+^NK1.1^−^CD4^−^ T cells, CD3^−^NK1.1^+^ NK cells and CD3^+^NK1.1^+^ NKT cells. Not surprisingly, IL-12, alone or in combination with IL-18, is known to induce IFN-γ production in T, NK and NKT cells and it appears that each of these cell types contributes to the reduction in IFN-γ production with the largest reduction in NK cell IFN-γ production (Fig. 9E) [37], [38], [39], [40]. Taken together, these data indicate that LAB facilitates IL-12 production from DCs and subsequent IFN-γ production mainly from NK and T cells *in vivo*. These data demonstrate that LAB is important for DC and NK, T and NKT cell-mediated cytokine production during the host response to *C. albicans* infection.

## Discussion

Here we identify for the first time an important role for LAB in anti-fungal immunity. *Lat2^−/−^* mice displayed increased susceptibility to *C. albicans* infection and the fungal burden was significantly increased in these mice, similar to *Clec4n^−/−^* mice and *Clec7a^−/−^* mice [6], [17]. *Lat2^−/−^* mice displayed reduced NK and T cell-mediated IFN-γ production which reflects the attenuated production of IL-12 by *Lat2^−/−^* DCs, while *Lat2^−/−^* neutrophil cytokine responses were not impaired. Moreover, we defined a novel role for LAB in the repression of β-catenin nuclear translocation in DCs, resulting in regulated cytokine production from a range of PAMPs, which is important in mounting an effective immune response in an infectious disease model. These data also suggest that LAB will be important in other diseases where IL-12 is involved in disease outcome.

The adaptor protein LAB mediates signals downstream of multiple ITAM-coupled receptors including BCR, FcRγ and TREM1/2 and the growth factor Stem Cell Factor [22]. LAB, similar to LAT, is targeted to lipid rafts where it nucleates signaling complexes [41]. Erk is activated downstream of ITAM-coupled receptors through the recruitment of a Grb2-Sos complex to LAT or LAB while PI3K is thought to be recruited through the formation of a LAB-Grb2-PI3K(p85) complex [22], [23]. Activation of these signaling pathways downstream of ITAM-coupled receptors results in cytokine production. However, while LAT and LAB can mediate cytokine production by facilitating formation of these signaling complexes, LAB has also been shown to negatively regulate cytokine production in certain circumstances. In some cases, this is due to competition with LAT which signals more efficiently than LAB due to the presence of a PLCγ binding site in LAT. Alternatively, in the absence of LAT, LAB binds the E3 ubiquitin ligase c-Cbl which targets multiple substrates for proteasomal degradation [23], [41]. LAB mediates the production of IL-12p40 and attenuates the production of IL-8/CXCL8, TNF and IL-10 downstream of LPS or ITAM-coupled receptors in macrophages and DCs [23], [24], [42]. Similarly, here we demonstrate that LAB partially mediates the production of IL-12p40, IL-12p70 and IL-23 in response to multiple PAMPs. Previous reports have shown that the levels of Th1 and Th17 inducing cytokines (IL-12, IL-23 and IL-1β) downstream of Dectin-1 and Dectin-2 are regulated by different NFκB subunits [43], [44]. However, while fungal and TLR-induced IL-12 family cytokine production is inhibited in the absence of LAB, IκB degradation is unaffected (Fig. S1 and data not shown). In addition, previously identified targets of LAB regulation such as Syk, c-Cbl or MAPK [23], [42] are also unaffected in the absence of LAB (Fig. S1). This suggests a role for LAB in the regulation of a distinct pathway.

Otero *et al* [32] recently identified an exciting link between M-CSF, DAP12 and β-catenin. The authors demonstrated that the ITAM-containing signaling chain DAP12 was required for the phosphorylation and accumulation of nuclear β-catenin during MCSF-induced signaling in macrophages. β-catenin is found in various locations in the cell. Under resting conditions, β-catenin is part of a complex found at the cell membrane bound to E-cadherin, which controls cell-cell adhesion [45]. In addition to membrane bound β-catenin, β-catenin is also found in the cytosol and cytosolic levels are tightly regulated by Wnt signaling. In the absence of a Wnt ligand β-catenin is phosphorylated by GSK-3β and CK1 and subsequently targeted for proteasomal degradation. In the presence of a Wnt ligand, GSK-3β activity is inhibited and β-catenin levels accumulate in the cytoplasm and the protein translocates to the nucleus to induce gene expression in conjunction with the TCF/LEF transcriptional activators [46]. Our data indicate that LAB is phosphorylated by two pathways, (M-CSF/DAP12 and Dectin-2) (Fig. S3), and that LAB inhibits basal β-catenin nuclear translocation mediated by the M-CSF/DAP12 pathway and also PAMP-induced β-catenin nuclear translocation. M-CSF is present in serum and organs of naïve mice and *C. albicans* infection further increases its levels [33] and provides the PAMPs necessary to induce these pathways *in vivo*. β-catenin and LAB have never before been associated and our data demonstrate that this is an important component of PAMP-induced cytokine production in DCs.

DCs expressing constitutively active β-catenin have previously been shown to display a diminished IL-12p40 response [35]. Additionally, ablation of β-catenin in DCs was recently shown to enhance the production of pro-inflammatory cytokines translating into increased Th1 and Th17 polarization [34]. Our results show that LAB plays a critical role in repression of the β-catenin pathway. In accordance with these findings, *Lat2^−/−^* DCs display high levels ofnuclear β-catenin accumulation and produce reduced levels of IL-12 resulting in reduced NK and T cell-mediated IFN-γ production. Activation of β-catenin by Wnt3a or SB-216763 results in a reduction of IL-12p40 production in WT DCs so that they now resemble those lacking LAB. Various PAMPs (LPS, Pam_3_CSK_4_, β-glucan) do not promote LAB phosphorylation, however, basal LAB phosphorylation appears to be sufficient to inhibit β-catenin activation and to facilitate PAMP-induced IL-12 production. The Dectin-2 pathway enhances LAB phosphorylation in response to various ligands (zymosan, *C. albicans*, CAWS) in addition to basal M-CSF/DAP12-induced LAB phosphorylation. These data indicate that basal LAB phosphorylation is sufficient to facilitate PAMP-induced IL-12 production and subsequent IFN-γproduction independently of Dectin-2. However, PAMPs that engage Dectin-2 likely play a cumulative role in promoting IL-12 production (Fig. S3). While the mechanism for LAB-mediated regulation of β-catenin activation is currently unknown, it is possible that LAB exerts its effects through regulation of the PI3K pathway. Akt phosphorylation promotes nuclear β-catenin accumulation [47] and we have previously shown in macrophages that LAB binds to the p85 subunit of PI3K, likely via Grb2 [23] suggesting that this may be a possible mechanism for LAB-mediated regulation of β-catenin translocation.

The LAB/β-catenin pathway in DCs exerts a specific effect on IL-12 family cytokines while minimally affecting other cytokines (Fig. 4E–F). There are some possible explanations for this specificity. Firstly, NFκB and Erk activation, critical components of IL-1β/TNF [48], [49] and IL-10 production, respectively [50] are normal in *Lat2^-/-^* DCs (Fig. S1). Secondly, β-catenin induces gene expression in conjunction with the TCF/LEF transcriptional activators [46] and examination of candidate TCF/LEF binding sites in the promoters of *Il12b, IL10* and *Tnf* revealed interesting differences. While *Il10* (−1 kb) and *Tnf* (−300 bp) contain one candidate TCF/LEF binding site, *Il12b* has two such sites ∼60 bp apart, ∼600 bp upstream of the transcription start site. Moreover an AML-1 binding site is located between these two TCF/LEF binding sites in *Il12b* and AML-1 has previously been shown to cooperate with TCF in the TCRá enhancer [51]. Thirdly, Jiang *et al* [45] demonstrated that cluster disruption of DCs, a process involving β-catenin, selectively increased IL-12p40 production in response to LPS (other cytokines such as TNF and IL-10 were reduced in response to LPS). All of these observations indicate a definite specificity of β-catenin for IL-12 family cytokines; however, separate studies would be required to dissect this.

Here we demonstrated a novel LAB-mediated pathway for regulation of IL-12 production and subsequent IFN-γ production in response to *C. albicans*. Collectively our data indicate the importance of this for controlling susceptibility to *C. albicans* infection. However, as LAB is expressed in other cell types, it may be involved in additional immune responses. For example, we have observed reduced IL-12p40 production from *Lat2^−/−^* macrophages (data not shown) similar to DCs suggesting that the reduced *Il12b* mRNA levels in *Lat2^−/−^* mice is likely a combined effect of DCs and macrophages. Additionally, neutrophils and associated cytokines (TNF, IL-6) are important for overcoming *C. albicans* infection [25], [52]. Interestingly, *Lat2^−/−^* neutrophils display enhanced TNF and IL-6 production, demonstrating unimpaired neutrophil activation and cytokine production in *Lat2^−/−^* mice. High levels of TNF and IL-6 are associated with septic shock [53], [54], however, the levels in *C. albicans-*infected *Lat2^−/−^* mice are much lower than those in murine sepsis models making it an unlikely cause for the increased mortality. In addition, LAB is expressed in B and activated T cells and its deficiency, similar to *Lat2^−/−^* neutrophils, results in “enhanced” rather than impaired responses. *Lat2^−/−^* mice have increased levels of natural antibodies and T cells from aged *Lat2^−/−^* mice are hyperactivated and produce more cytokine than WT T cells [55]. These data indicate that reduced DC/macrophage IL-12 production and subsequent NK and T cell-mediated IFN-γ production is the predominant impairment found in *Lat2^−/−^* mice, however, the role of LAB in other cell-types and functions during anti-fungal immunity needs to be addressed with future studies.

In conclusion, we have shown that LAB is paramount for robust pro-inflammatory cytokine production from DCs downstream of multiple PAMPs. LAB facilitates production of these essential cytokines through novel regulation of the β-catenin pathway. LAB represses β-catenin nuclear accumulation in DCs, thereby facilitating cytokine production. Through this mechanism, LAB plays an important role in the host defense against systemic *C. albicans* infection by inducing NK and T cell-mediated IFN-γ production. These data are the first demonstration of LAB as a prominent target for diseases dependent on IL-12 and further elucidation of these pathways may be important for the development of new therapeutics.

## Materials and Methods

### Mice

*Lat2^−/−^* mice (the product of the *Lat2* locus is the adaptor protein LAB), described previously [56], have been backcrossed onto the C57BL/6 background for at least 10 generations. These mice were screened and found to be 99% C57BL/6. *Lat2^−/−^, Myd88^−/−^, Fcer1g^−/−^, Tyrobp^−/−^* and age, weight and gender matched control C57BL/6 mice were maintained under specific pathogen-free conditions at the NCI–Frederick, MD. Animal care was provided in accordance with the procedures in, “*A Guide for the Care and Use of Laboratory Animals”*. Ethical approval for the animal experiments detailed in this manuscript was received from the Institutional Animal Care and Use Committee (Permit Number: 000386) at the NCI-Frederick.

### BMDC culture

Bone marrow (BM) cells were removed from the femurs and tibiae of mice and erythrocytes were lysed in ACK buffer. Bone marrow-derived dendritic cells (BMDCs) were generated by culturing cells for 6–9 days in RPMI 1640 medium containing 10% fetal bovine serum, 2 mM L-glutamine, penicillin/streptomicin, HEPES, NEAA, Sodium pyruvate, 2-mercaptoethanol and 10 ng/ml GM-CSF.

### Reagents and antibodies

Zymosan, particulate β-glucan, LPS (*Escherichia* coli 0111:B4) and purified mannan were purchased from Sigma-Aldrich (St. Louis, MO). CAWS (*C. albicans* water soluble mannans) were provided by Prof. Naohito Ohno [6]. Pam_3_CSK_4_ was purchased from Invivogen (San Diego, CA), Wnt3a and anti-CD3 (clone 145-2C11) were purchased from R&D (Minneapolis, MN). Anti-CD28 (37.51), anti-CD4 (RM4.5), anti-CD25 (PC61.5), anti-CD44 (IM7), anti-CD62L (MEL-14), anti-Ly6G (1A8), anti-CD11b (M1/70), anti-IL-6 (MP5-20F3), anti-IFN-γ (XMG1.2) and anti-IL-17A (TC11-18H10.1) were purchased from BD Biosciences (San Jose, CA). Anti-TNF (MP6-XT22) was purchased from eBioscience (San Diego, CA). GM-CSF, M-CSF and M-CSF ELISA were purchased from Peptotech (Rocky Hill, NJ). The GSK-3β inhibitor SB-216763 and the Tankyrase inhibitor XAV939 were purchased from Tocris Bioscience (Ellisville, MO). The Syk inhibitor Piceatannol was purchased from Millipore (Billerica, MA). Anti-phosphoErk, anti-Erk, anti-phosphoAkt, anti-Akt, anti-β-catenin were purchased from Cell Signaling Technology (Beverley, MA). Anti-LAT antibody [28] was as previously described. Anti-phosphotyrosine (clone 4G10, Millipore), anti-Syk (Novus Biologicals, Littleton, CO), anti-LAB, anti-c-Cbl (sc-170), anti-PLCγ (Santa Cruz, CA), anti-Actin (Chemicon International, Temecula, CA), anti-Lamin B1 (Abcam, Cambridge, MA) and anti-Dectin-2 (AbD Seotech, Raleigh, NC) were used in this study.

### Reagent preparation

Zymosan was resuspended in 10% ethanol in endotoxin free water. Zymosan extract was prepared by resuspending zymosan in endotoxin free water. The supernatant was filtered and used as zymosan extract. The zymosan particles were washed 3× with endotoxin free water and used as washed zymosan.

### Cell purification and stimulations

BM cells were removed from the femurs and tibiae of mice and erythrocytes were lysed in ACK buffer. Neutrophils were purified through percoll gradient or by MACS separation (Miltenyi, Auburn, CA). BMDCs were harvested and CD11c^+^ DCs were sorted using a FACS ARIA or bead purified by MACS separation routinely giving purities of >98%. BMDCs were serum starved for 30 min at 37°C (except for Figs 7A&D). 1×10^7^ BMDCs were resuspended in 100 µl DPBS and stimulated at 37°C with 1 mg/ml zymosan, zymosan extract, washed zymosan, β-glucan, mannan, Pam_3_CSK_4_, 10 ng/ml LPS, 100 ng/ml GM-CSF or M-CSF for the indicated times. Cells were lysed with Lauryl-maltoside lysis buffer (1% laurylmaltoside in 20 mM Tris [pH 7.5], 100 mM NaCl, 10% glycerol, 0.4 mM Na_3_VO_4_, aprotinin, leupeptin and phenylmethylsulfonyl fluoride). Lysates were clarified by centrifugation and protein levels were normalized using a BCA protein assay. Cytosol and nuclear extracts were prepared as previously described [57]. 4× non-reducing or reducing Nupage sample buffer was added to lysates and heated for 10 min at 70°C. Lysates were separated by SDS-PAGE (Nupage, Invitrogen, Carisbad, CA), transferred to PVDF membrane (Millipore, Billerica, MA) and analyzed by Western blot.

### RNA isolation and quantitative RT-PCR

Cells were resuspended in Trizol and RNA was extracted using RNeasy Mini Kit (Qiagen, Valencia, CA). cDNA was synthesized from total RNA using Superscript III First Strand Synthesis System for RT-PCR (Invitrogen). Quantitative RT-PCR was performed using ABI Taqman Primer and Probe sets and normalization was performed against *Hprt1*.

### Lentiviral knockdown

An shRNA construct for mouse Dectin-2 (TRCN0000066785) and a scrambled shRNA control in the pLKO.1 lentiviral vector were used to infect WT and *Lat2^−/−^* cells. 293FT cells were transfected with the pLKO.1 construct and with the Invitrogen packaging constructs pLP1, pLP2 and pVSV-G and viral supernatants were collected at 48 and 72 h post-transfection. BM cells were plated on Day 0 at 6×10^5^ cells/ml in complete media containing GM-CSF. On Days 1 and 2, the media was replaced for 8 h with viral supernatants containing 10 µg/ml hexamethrine bromide. On Day 3, fresh complete media containing GM-CSF and 5 µg/ml puromycin was added to the cells. The cells were harvested 2 days later and stimulated as described. Dectin-2 surface expression was examined by flow cytometric analysis.

### Cytokine assays

BMDCs were plated at a density of 1×10^5^ cells/well in a 96-well plate in RMPI containing 10% fetal bovine serum and 10 ng/ml GM-CSF. BMDCs were stimulated with zymosan, zymosan extract, washed zymosan, Pam_3_CSK_4_, LPS (*Escherichia* coli 0111:B4), β-glucan, curdlan, yeast/hyphae or heat-killed yeast/hyphae for 24 h. BMDCs were stimulated with CAWS for 48 h. BM neutrophils were plated at a density of 2×10^5^ cells/well in RPMI containing 10% fetal bovine serum and stimulated with zymosan or LPS for 24 h. Cell culture supernatants were recovered and assayed for cytokine by ELISA (eBioscience, San Diego, CA) or cytometric bead array (CBA) (BD Biosciences, San Jose, CA), according to the manufacturer's protocol. Unpurified spleen cells or CD11c^+^ MACS bead purified spleen cells were stimulated with 100 ng/ml LPS for 6 h in the presence of Brefeldin A. IL-12p40 producing cells were determined by flow cytometry.

### T cell polarization assay

CD4^+^ T cells were purified from WT spleens by negative selection MACS separation (Miltenyi, Auburn, CA) and naïve CD4^+^ T (CD4^+^CD25^−^CD44^lo^CD62L^+^) cells were sorted using a FACS ARIA giving purities of >98% purity. Naïve CD4^+^ T cells were stimulated with plate-bound anti-CD3 and soluble anti-CD28 antibodies for 4 days and supplemented with conditioned supernatants from BMDCs stimulated with heat-killed *C. albicans* yeast or LPS. The supernatants were collected and analyzed for IL-17A and IFN-γ production by ELISA. The cells were restimulated with PMA (50 ng/ml) and Ionomycin (0.5 µg/ml) in the presence of monensin (3 µM) and IL-17A and IFN-γ producing cells were determined by flow cytometry.

### *In vivo* model of systemic candidasis

*C. albicans* SC5314 (ATCC, Manassas, VA) was cultured for 24 h in YEPD broth, washed three times with PBS and resuspended at the required concentration in PBS. Mice were matched by gender, weight and age (10–15 weeks old) and 100 µl of *C. albicans* in PBS was injected i.v. Mice were monitored and weighed daily. Mice were euthanized by CO_2_ asphyxiation when they were moribund or had lost 20% of their body weight. Experiments were continued for a maximum of 55 days at which point all surviving mice were euthanized. Mice were bled by cardiac puncture after CO_2_ administration and kidneys and spleens were harvested. The kidneys were either placed in 10% formalin, embedded in paraffin wax blocks and stained for H&E and PAS or they were placed in PBS, dounce homogenized and serial dilutions were plated on YEPD agar containing 50 µg/ml chloramphenicol. The plates were cultured for 24 h and CFU were calculated/organ. The spleens were dounce homogenized in trizol followed by RNA and cDNA preparation. Serum samples were analyzed by CBA for cytokine levels.

### *Ex vivo C.albicans* NK and T cell assay

Mice were injected intraperitoneally with 1×10^5^ live *C. albicans* and euthanized 72 h later. The inflammatory infiltrate was collected by peritoneal lavage with 5 ml 5 mM EDTA in RPMI. The cells were plated and restimulated with PMA (50 ng/ml) and Ionomycin (0.5 µg/ml) in the presence of monensin (3 µM) for 4 h and IL-17A and IFN-γ producing cells were determined by flow cytometry.

### *C. albicans* neutrophil recruitment and cytokine production

Mice were injected intraperitoneally with 1×10^5^ live *C. albicans* and euthanized 4 h later. The inflammatory infiltrate was collected by peritoneal lavage with 2 rounds of 5 ml 5 mM EDTA in RPMI. The cells were plated and restimulated with media or heat-killed *C. albicans* yeast for 12 h in the presence of monensin (3 µM). The % of TNF and IL-6 producing neutrophils were determined by flow cytometry.

### Statistical methods

Data are presented as means +/− s.e.m. and are representative of 2–3 independent experiments. Survival data was analyzed by log-rank test. One-way ANOVA followed by Bonferroni's post-test or two-way ANOVA were used for statistical analysis when multiple groups were analyzed. Student *t* test or Mann-Whitney test were used for statistical analysis when two groups were analyzed. When data did not follow a Gaussian distribution, it was transformed by Y = sqrt(Y+0.5) [58] and analyzed by Student's *t* test. If data still did not follow a Gaussian distribution after transformation, then significance was tested by Mann-Whitney test. Statistical significance was set at *p<0.05 **p<0.005 ***p<0.0005.

## Supporting Information

Figure S1 **Normal signaling in** ***Lat2^−/−^*** **BMDCs.** (A–D) BMDCs from WT and *Lat2^−/−^* mice were stimulated with 1 mg/ml zymosan extract (A–C) or 1 mg/ml zymosan (D) for the indicated times. (A) WCL were immunoblotted with anti-phosphotyrosine, anti-LAB and anti-actin. (B) Cells were immunoprecipitated with anti-Syk, anti-c-Cbl, and anti-PLCγ2 and immunoblotted with anti-phosphotyrosine, anti-Syk, anti-c-Cbl, and anti-PLCγ2. (C) WCL were immunoblotted with anti-phospho-Erk and anti-Erk. (D) WCL were immunoblotted with anti-IκB and anti-actin.
(TIF)

Figure S2 **IL-12p40 and IFN-γ production is reduced in** ***Lat2^−/−^*** **cells** ***ex vivo*** **.** (A) Splenic cells from WT and *Lat2^−/−^* mice were stimulated with 100 ng/ml LPS for 6 h. IL-12p40 levels in CD11c^+^MHCII^+^ DCs were measured by flow cytometry. Plots are representative of 4 mice and data are representative of 2 independent experiments. (B–C) WT and *Lat2^−/−^* mice were injected intraperitoneally with *C. albicans*. Cells were collected by peritoneal lavage 72 h post injection and re-stimulated with PMA/Ionomycin. IFN-γ and IL-17 producing NK1.1^−^CD3^+^CD4^+^ T cells (B) and NK1.1^+^CD3^−^ NK cells (C) were measured by flow cytometry. Plots are representative of 6 mice and data are representative of 2 independent experiments.
(TIF)

Figure S3 **Model of LAB involvement in IL-12 production.** (1) M-CSF is recognized by the M-CSFR on dendritic cells. Through crosstalk with DAP12, M-CSF promotes LAB phosphorylation. LAB inhibits β-catenin translocation to the nucleus, thereby promoting IL-12 following subsequent stimulation with PAMPs. M-CSF/DAP12-induced LAB phosphorylation is sufficient to inhibit β-catenin activation and subsequent PAMP-induced IL-12 production. In *Lat2^−/−^* DCs, basal β-catenin levels are increased. (2) *C. albicans*, zymosan or CAWS stimulate the Dectin-2 pathway, further stimulating LAB phosphorylation and inhibiting nuclear translocation of β-catenin. These fungal PAMPs also induce IL-12 production. In *Lat2^−/−^* DCs, IL-12 production is reduced. (3) β-glucan, LPS or Pam_3_CSK_4_ stimulate IL-12 production but they do not stimulate LAB phosphorylation. In *Lat2^−/−^* DCs, IL-12 production is reduced demonstrating that basal M-CSF/DAP12-induced LAB phosphorylation is sufficient to control β-catenin levels and IL-12 production. (4) LAB-mediated IL-12 produced by DCs promotes IFN-γ production from NK, T and NKT cells. IFN-γ production is reduced in the absence of LAB.
(TIF)
