# Supplementary figures and images for: LAB/NTAL Facilitates Fungal/PAMP-induced IL-12 and IFN-γ Production by Repressing β-Catenin Activation in Dendritic Cells
Source: PLoS Pathog. 2013 May 9;9(5):e1003357. doi: 10.1371/journal.ppat.1003357 (PMC3649983; doi:10.1371/journal.ppat.1003357)

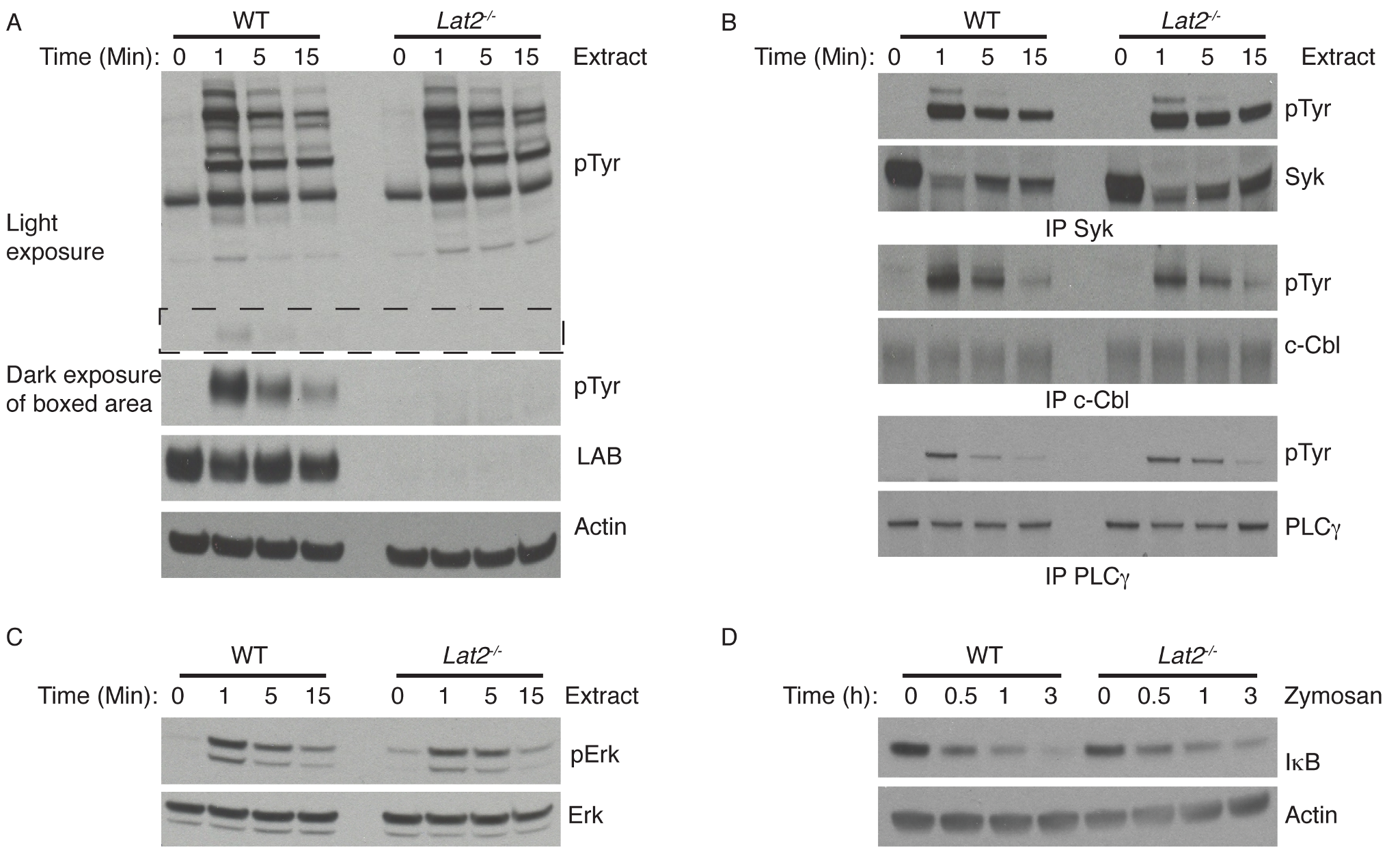

Supplement: Figure S1 — Normal signaling in Lat2−/− BMDCs. (A–D) BMDCs from WT and Lat2−/− mice were stimulated with 1 mg/ml zymosan extract (A–C) or 1 mg/ml zymosan (D) for the indicated times. (A) WCL were immunoblotted with anti-phosphotyrosine, anti-LAB and anti-actin. (B) Cells were immunoprecipitated with anti-Syk, anti-c-Cbl, and anti-PLCγ2 and immunoblotted with anti-phosphotyrosine, anti-Syk, anti-c-Cbl, and anti-PLCγ2. (C) WCL were immunoblotted with anti-phospho-Erk and anti-Erk. (D) WCL were immunoblotted with anti-IκB and anti-actin. (TIF) [file ppat.1003357.s001.tif]

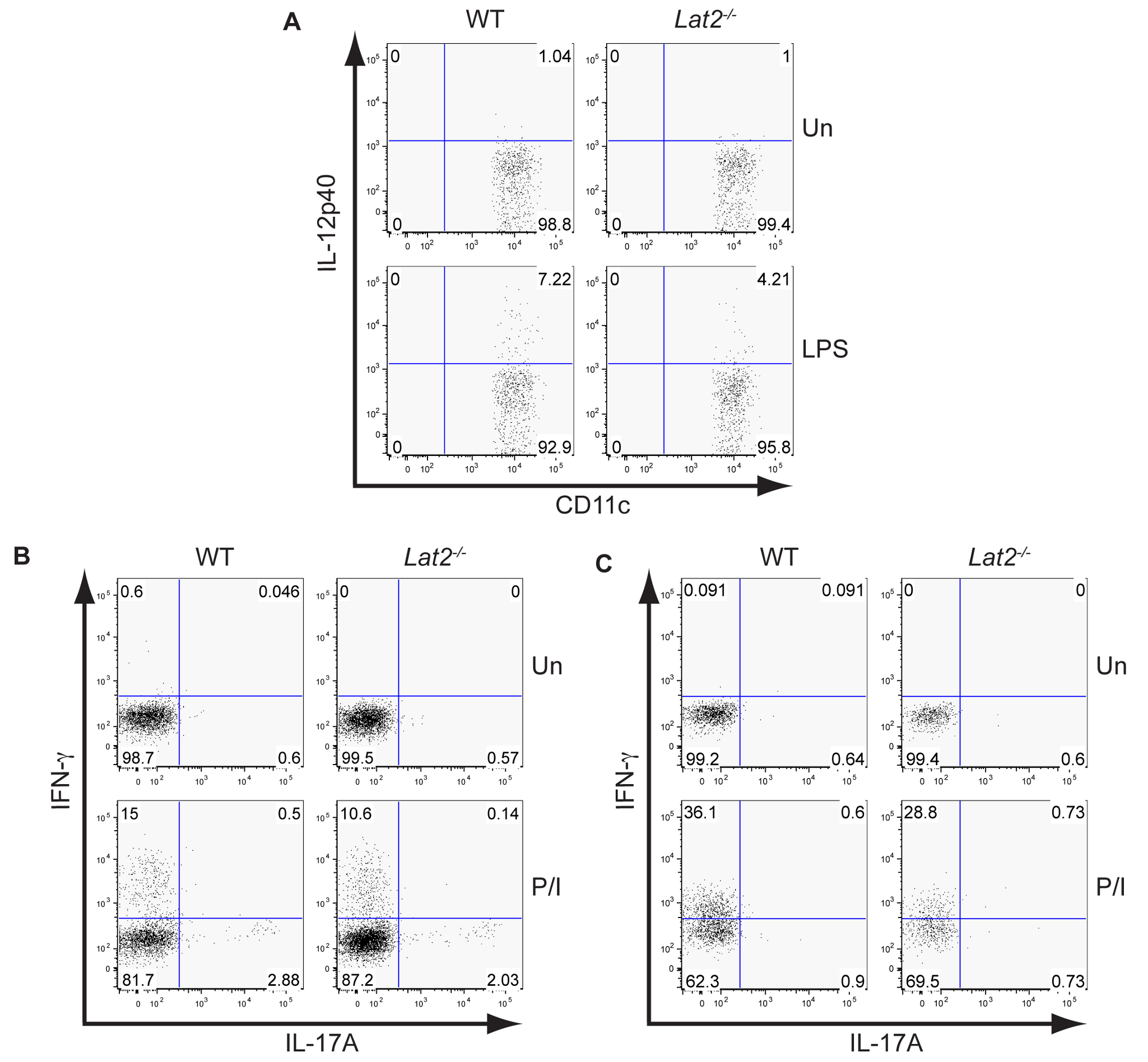

Supplement: Figure S2 — IL-12p40 and IFN-γ production is reduced in Lat2−/− cells ex vivo . (A) Splenic cells from WT and Lat2−/− mice were stimulated with 100 ng/ml LPS for 6 h. IL-12p40 levels in CD11c+MHCII+ DCs were measured by flow cytometry. Plots are representative of 4 mice and data are representative of 2 independent experiments. (B–C) WT and Lat2−/− mice were injected intraperitoneally with C. albicans. Cells were collected by peritoneal lavage 72 h post injection and re-stimulated with PMA/Ionomycin. IFN-γ and IL-17 producing NK1.1−CD3+CD4+ T cells (B) and NK1.1+CD3− NK cells (C) were measured by flow cytometry. Plots are representative of 6 mice and data are representative of 2 independent experiments. (TIF) [file ppat.1003357.s002.tif]

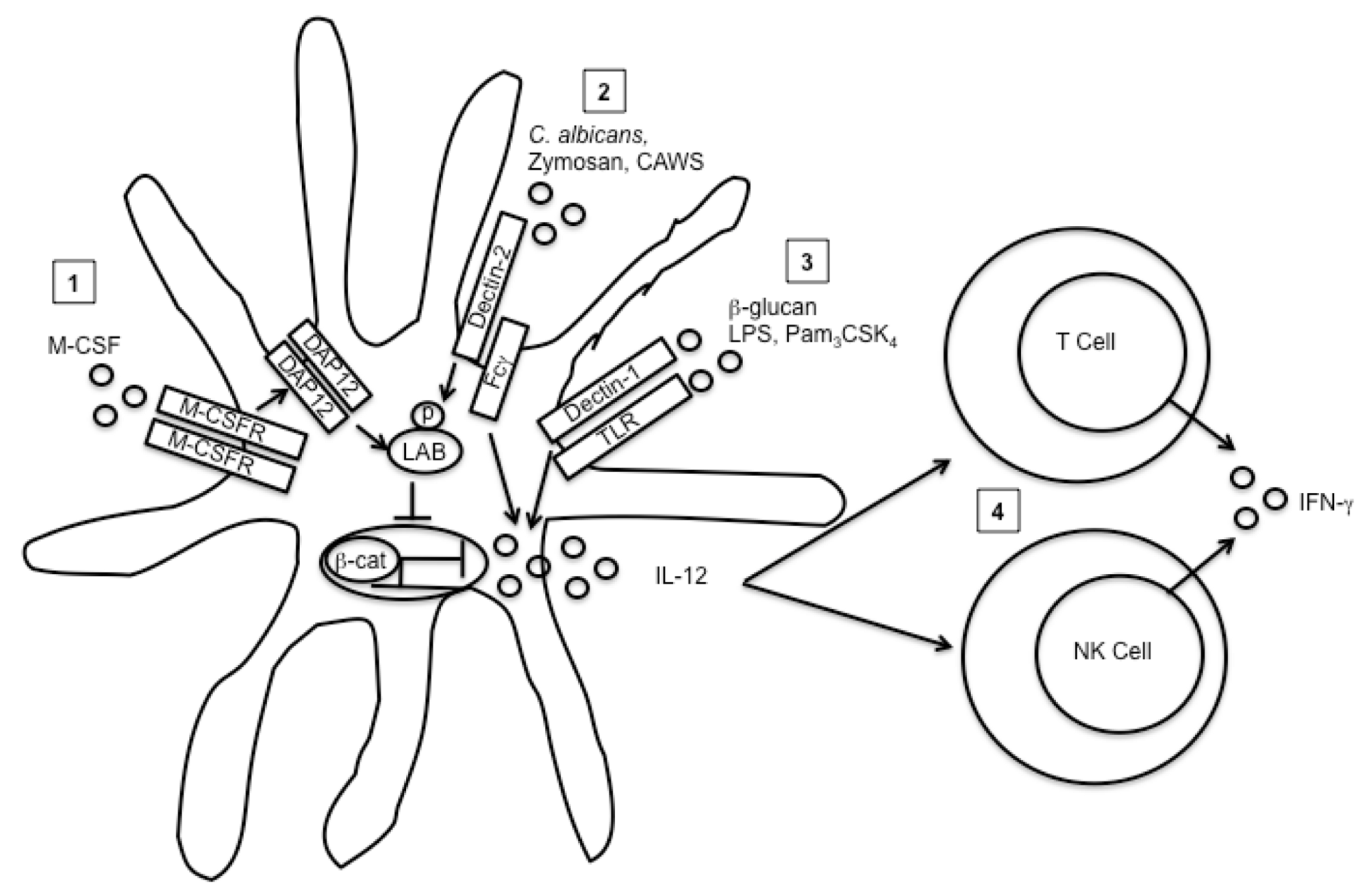

Supplement: Figure S3 — Model of LAB involvement in IL-12 production. (1) M-CSF is recognized by the M-CSFR on dendritic cells. Through crosstalk with DAP12, M-CSF promotes LAB phosphorylation. LAB inhibits β-catenin translocation to the nucleus, thereby promoting IL-12 following subsequent stimulation with PAMPs. M-CSF/DAP12-induced LAB phosphorylation is sufficient to inhibit β-catenin activation and subsequent PAMP-induced IL-12 production. In Lat2−/− DCs, basal β-catenin levels are increased. (2) C. albicans, zymosan or CAWS stimulate the Dectin-2 pathway, further stimulating LAB phosphorylation and inhibiting nuclear translocation of β-catenin. These fungal PAMPs also induce IL-12 production. In Lat2−/− DCs, IL-12 production is reduced. (3) β-glucan, LPS or Pam3CSK4 stimulate IL-12 production but they do not stimulate LAB phosphorylation. In Lat2−/− DCs, IL-12 production is reduced demonstrating that basal M-CSF/DAP12-induced LAB phosphorylation is sufficient to control β-catenin levels and IL-12 production. (4) LAB-mediated IL-12 produced by DCs promotes IFN-γ production from NK, T and NKT cells. IFN-γ production is reduced in the absence of LAB. (TIF) [file ppat.1003357.s003.tif]
